# Interesse als Katalysator: Die Rolle des Interesses bei der Nutzung gestufter Lernhilfen im Forschenden Lernen

**DOI:** 10.1007/s40573-026-00197-y

**Published:** 2026-10-16

**Authors:** Julia Arnold, Andrea Denise Lüscher, Charlotte Schneider, Andrea Maria Schmid

**Affiliations:** 1 Zentrum Naturwissenschafts- und Technikdidaktik (ZNTD), Pädagogische Hochschule FHNW, Hofackerstrasse 30, 4132 Muttenz, Schweiz; 2 Institut für Fachdidaktik MINT und Nachhaltigkeit, PH Luzern, Luzern, Schweiz

**Keywords:** Situationales Interesse, Forschendes Lernen, Gestufte Lernhilfen, Lernzielorientierung, Fähigkeitsselbstkonzept, Situational interest, Inquiry-based learning, Incremental scaffolds, Mastery goal orientation, Ability self-concept

## Abstract

Der vorliegende Beitrag untersucht die Rolle des situationalen Interesses im naturwissenschaftlichen Unterricht, insbesondere im Zusammenhang mit der Nutzung gestufter Lernhilfen in einer digitalen Lernumgebung zum Experimentieren (forschendes Lernen). Theoretisch fundiert auf der Person-Gegenstands-Theorie des Interesses wird analysiert, wie Interesse das Fähigkeitsselbstkonzept und die persönliche Lernzielorientierung beeinflusst und wie diese Faktoren die Nutzung von Lernhilfen und den Lernerfolg determinieren. In einer Interventionsstudie mit Schüler:innen im Alter von 13 bis 19 Jahren (*N* = 480) wurde gezeigt, dass höheres situationales Interesse positiv auf die Lernzielorientierung und die Wahl instrumenteller Lernhilfen (Hinweise und Beispiele) wirkt, was zu einem signifikanten Lernzuwachs führt. Dabei zeigt sich ein kleiner Effekt des Geschlechts: Mädchen weisen tendenziell ein höheres Interesse auf, während Jungen ein stärkeres Fähigkeitsselbstkonzept berichten. Die Ergebnisse unterstützen die Annahme, dass das situationale Interesse in Verbindung mit einem positiven Fähigkeitsselbstkonzept die Nutzung lernförderlicher Hilfen begünstigt und somit den Lernerfolg steigert. Der Beitrag liefert wertvolle Einblicke in die Bedeutung des Interesses im naturwissenschaftlichen Unterricht und bietet Implikationen für die gezielte Förderung von Interesse, insbesondere zur Unterstützung von Schülerinnen in naturwissenschaftlichen Fächern.

## Abstract (en)

In this article we examine the role of situational interest in science education, particularly in relation to the use of incremental scaffolds in a digital learning environment for experimentation (inquiry-based learning). Theoretically based on the person-object theory of interest, we analyze how interest influences the ability self-concept and personal mastery goal orientation and how these factors determine the use of scaffolds and learning success. In an intervention study with students aged 13 to 19 years (*N* = 480), we showed that higher situational interest has a positive effect on mastery goal orientation and the choice of instrumental scaffolds (hints and examples), which leads to a significant increase in learning. There was a small effect of gender: girls tended to show a higher level of interest, while boys reported a stronger ability self-concept. The results support the assumption that situational interest in conjunction with a positive ability self-concept favors the use of learning-promoting use of scaffolds and thus increases learning success. The article provides valuable insights into the importance of interest in science education and offers implications for the targeted promotion of interest, especially to support female students in science subjects.

## Einleitung

Interesse ist essenziell im naturwissenschaftlichen Unterricht. Interesse kann sowohl die Motivation der Lernenden, sich mit den Inhalten auseinanderzusetzen, positiv beeinflussen als auch ihr Lernen. Beispielsweise zeigen interessierte Schüler:innen (S:S) bessere Leistungen in Tests und sind häufiger bereit, sich freiwillig und intensiv mit dem Lernstoff zu beschäftigen (Potvin und Hasni 2014). Nicht zuletzt bildet Interesse die Grundlage für die in den naturwissenschaftlichen Fachdidaktiken weit verbreitete Kontextorientierung (für eine Übersicht: Zügge 2022). Darüber hinaus kann Interesse Disparitäten im naturwissenschaftlichen Unterricht verringern, indem es die Motivation und das Engagement für naturwissenschaftliche Themen steigert (Bennett et al. 2007; Habig et al. 2025; Ültay und Çalık 2012).

Im selbstregulierten Lernen, wie bspw. offenen Formen des forschenden Lernens, kommt dem Interesse eine besondere Funktion zu. Die Lernenden müssen sich nicht nur für die Auseinandersetzung mit dem Stoff motivieren. Im Vergleich zu eher angeleitetem Unterricht müssen sie sich viel stärker auch mit der Nutzung spezifischer Strategien wie bspw. der Hilfen-Nutzung auseinandersetzen. Dabei steht das Interesse mit weiteren motivationalen Faktoren wie dem Fähigkeitsselbstkonzept und der Zielorientierung in Zusammenhang. Im vorliegenden Beitrag werden diese Zusammenhänge in Bezug auf die Nutzung von gestuften Lernhilfen beim forschenden Lernen untersucht und damit verbunden auch die Auswirkung auf den Erwerb von Experimentierkompetenzen.

## Theoretischer Hintergrund & Forschungsstand

### Experimentierkompetenz und Forschendes Lernen

Naturwissenschaftlicher Unterricht muss grundlegendes Fachverständnis sowie Kompetenzen der Erkenntnisgewinnung als Teil der naturwissenschaftlichen Grundbildung fördern. Lernende sollen daher die Fähigkeit erlernen, naturwissenschaftliche Untersuchungen durchzuführen und zu beurteilen. Diese Fähigkeit wird unterschiedlich konzeptualisiert (Gut-Glanzmann und Mayer 2018) und als Teil des wissenschaftlichen Denkens verstanden (Mayer 2007). Das Experiment ist die zentrale Methode zur Untersuchung kausaler Zusammenhänge (Wellnitz und Mayer 2013) und sollte laut einer Delphi-Studie von S:S beherrscht werden (Osborne 2003). Es steht daher im Zentrum dieser Studie. Unterschiedliche Literaturquellen diskutieren Teilkompetenzen von der Identifizierung von Forschungsfragen bis zur Datenaufbereitung und -präsentation (Gut-Glanzmann 2012; Nehring et al. 2016; Rönnebeck et al. 2016; Wellnitz et al. 2017). Ein gemeinsames Ziel ist, dass S:S Untersuchungen planen und durchführen können, womit das Planen von Experimenten eine wichtige Teilkompetenz darstellt, die im Zentrum dieses Beitrags steht (Arnold et al. 2018; Schiepe-Tiska et al. 2016). Beispielsweise sind das Erkennen und Operationalisieren der abhängigen Variable oder das Bestimmen und Variieren der unabhängigen Variable gezielt zu fördern (Wellnitz und Mayer 2013).

Zur Förderung dieser Kompetenzen eignet sich die Unterrichtsmethode des forschenden Lernens (Chinn und Malhotra 2002). Forschendes Lernen ist ein konstruktivistischer Ansatz, der den Prozess der naturwissenschaftlichen Erkenntnismethode abbildet und besonders zur Förderung der Experimentierkompetenz geeignet ist (Abd-El-Khalick et al. 2004; Hmelo-Silver et al. 2007). Offenere Ansätze, wie das eigenständige Experimentieren, können jedoch für unerfahrene S:S überfordernd sein (Kirschner et al. 2006) und Frustration hervorrufen (Sadeh und Zion 2009). Daher kommt Lernunterstützungen eine bedeutende Rolle zu (Lazonder und Harmsen 2016).

Die optimale Form dieser Unterstützung und die Rolle der Lehrpersonen sind Gegenstand von Forschung (Zion und Mendelovici 2012). Eine explizite Förderung der Experimentierkompetenz durch Hinweise und Handlungsanweisungen kann lernförderlich sein (Vorholzer et al. 2020). Es ist jedoch eine Herausforderung, Unterstützung zu geben, ohne den selbstregulierten Charakter des Unterrichts zu unterminieren (Wiggins und McTighe 2005). Standardisierte Formate zur Unterstützung des forschenden Lernens, wie gestufte Lernhilfen, empfehlen sich daher.

### Gestufte Lernhilfen

Gestufte Lernhilfen leiten sich aus Beispielaufgaben (*worked examples*) ab. Beispielaufgaben bestehen aus einem kontextualisierten Problem, Lösungsschritten und der finalen Lösung, die Schritt für Schritt in vorgegebener Reihenfolge durchgearbeitet werden (Atkinson et al. 2000; Renkl 2005; Renkl und Atkinson 2010). Zwar können bei Beispielaufgaben einzelne Lösungschritte ausgelassen werden (*fading*), es findet jedoch keine Diagnose und entsprechend keine diagnose-basierte Anpassung der Unterstützung auf das Lernniveau im Sinne des selbstregulierten Lernens statt (Puntambekar und Hubscher 2005). Die Wirksamkeit von Beispielaufgaben gegenüber dem offenen Problemlösen wurde in der Meta-Analyse von Crissman (2006) untersucht, die Studien in der Sekundarstufe über verschiedene Disziplinen hinweg analysiert. Es zeigte sich eine mittlere Effektstärke der Beispielaufgaben in Bezug auf den Lernzuwachs im Fachwissen gegenüber offenen Problemlöseaufgaben. Als besonders wirksam konnte hier das schrittweise Reduzieren von Lösungsschritten identifiziert werden. Darüber hinaus liegt bereits eine Studie vor, die die Wirksamkeit von Lösungsbeispielen für den Erwerb naturwissenschaftlicher Denk- und Arbeitsweisen in der sechsten Schulstufe untersucht (Koenen et al. 2017). Die Ergebnisse weisen jedoch darauf hin, dass experimentelle Problemlöseaufgaben lernwirksamer sind als das Arbeiten mit Beispielaufgaben.

Bei gestuften Lernhilfen werden im Gegensatz zu Beispielaufgaben Lösungshinweise einzeln gegeben und die Lernenden können nach Bedarf darauf zurückgreifen. Somit wird die Diagnose, die bei den ursprünglichen Beispielaufgaben nicht gegeben ist, in die Hand der Lernenden gegeben (Schmidt-Weigand et al. 2008). Dieses *on-demand*-Vorgehen hat den Vorteil, dass keine unnötigen oder unpassenden Unterstützungen angeboten werden, und dass der optimale Zeitpunkt von den Lernenden selbst bestimmt und so günstig für die Wissenskonstruktion genutzt werden kann (Aleven et al. 2003). Zudem ist es möglich, dass die Lernenden mit anwachsendem Fähigkeitsniveau die Unterstützungen selbst reduzieren und zunehmend selbständiger werden. Im Bereich naturwissenschaftlicher Problemlöseaufgaben haben sich gestufte Lernhilfen gegenüber konventionellen, als Ganzes dargebotenen Lösungsbeispielen bezüglich deklarativen Wissens (Fachwissen) als lernwirksamer erwiesen (Schmidt-Borcherding et al. 2013; Schmidt-Weigand et al. 2008).

In einer früheren Studie wurden die gestuften Lernhilfen, die aus Hinweisen (instrumentelle Hilfe) und Lösungen (exekutive Hilfe) bestehen, mit dem Experimentieren in Verbindung gebracht und ihre Lernförderlichkeit für Kompetenzen der Erkenntnisgewinnung nachgewiesen (Arnold 2015; Arnold et al. 2017). Um die optimale Lernunterstützung zu erhöhen, wurden für die vorliegende Studie die Hilfen mit Beispielen auf der Grundlage der Bloomschen Taxonomie (Krathwohl 2002) ergänzt: 1) Hinweise, die wesentliche Begriffe und Detailinformationen verdeutlichen (instrumentelle Hilfe), 2) Beispiele, die das Vorgehen anhand eines anderen Beispiels beschreiben (instrumentelle Hilfe) und 3) Lösungen, die den Lernenden Lösungsschritte und die endgültige Lösung vorgeben (exekutive Hilfe). Die Lernenden können diese *on-demand*-Hilfe nach Belieben und entsprechend ihren Bedürfnissen nutzen.

### Mal-adaptive Hilfen-Nutzung und mögliche Ursachen

Ein Nachteil von *on-demand-*Hilfen, wie es die gestuften Lernhilfen sind, ist, dass die S:S durch die freiwillige Hilfen-Nahme die Möglichkeit erhalten, die Hilfen nicht angemessen zu nutzen. Aleven et al. (2003) unterscheiden zwei Arten von mal-adaptiver Hilfen-Nutzung: Um Hilfen-Missbrauch handelt es sich, wenn Hilfen unnötig oder nicht angemessen genutzt werden, z. B. wenn zu wenig Zeit mit Lösungsschritten verbracht und direkt zur Lösung übergegangen wird. Außerdem werden bei mal-adaptiver Nutzung vermehrt exekutive Hilfen, die z. B. die Lösung vorgeben, gesucht. Diese helfen in der Regel nur kurzfristig und stehen im Kontrast zu instrumentellen Hilfen (z. B. Hinweise), die das selbständige Arbeiten weiterbefördern und langfristig lernförderlich sein können (Nelson-Le Gall 1985). Um Hilfen-Vermeidung handelt es sich, wenn trotz Bedarf keine Hilfe in Anspruch genommen wird (z. B. Wood und Wood 1999). Für diese Verhaltensweisen kommen motivationale Faktoren wie Interesse, Fähigkeitsselbstkonzept und Zielorientierung in Betracht.

### Interesse

Die in der fachdidaktischen Forschung der Naturwissenschaften weit verbreitete *Person-Gegenstands-Theorie des Interesses* definiert Interesse als dynamischen Prozess zwischen einer Person und einem Objekt und berücksichtigt individuelle und umweltbedingte Faktoren sowie deren Interaktion (Hidi und Renninger 2006; Krapp 1999; Krapp und Prenzel 2011). Interesse kann als Teil des Konstrukts intrinsischer Motivation eingeordnet werden, wobei Interesse selbst immer auf ein bestimmtes Objekt fokussiert. Es äußert sich im Verhalten durch Merkmale wie Aufmerksamkeit und Orientierung auf etwas oder jemanden (ebd.). Es wird zwischen zwei Formen von Interesse unterschieden: Situationales Interesse (a) tritt während der Auseinandersetzung mit einem Objekt in einer bestimmten Situation auf. Von individuellem oder auch dispositionalem Interesse (b) wird dann gesprochen, wenn es als Internalisierung in einen längeren anhaltenden Zustand überführt wurde. Individuelles Interesse ist eher stabil als persönliche Disposition einzuordnen und situationales Interesse eher kurzfristig, an aktuelle Gegenstände bzw. Inhalte und Aktivitäten geknüpft. In der vorliegenden Studie ist das situationale Interesse im Fokus, da davon ausgegangen werden kann, dass dieses am stärksten mit den momentanen Handlungen (bspw. Nutzung von Hilfen) zusammenhängt.

Gardner und Tamir (1989) schlagen ein multidimensionales Konzept von Interessensmessung vor. Krapp und Prenzel (2011) differenzieren in Anlehnung an Häussler (1987) die drei Dimensionen Thema, Kontext und Aktivität. Des Weiteren beschreiben Schiefele et al. (1993) das Interesse als bestehend aus einer wertbezogenen („ich finde es wichtig“) und einer emotionalen Komponente („es macht mir Spaß“), die Valenzen (bspw. persönliche Bedeutsamkeit oder Freude) mit dem Interessensgegenstand verknüpfen. Der intrinsische Charakter des Interesses („ich würde mich auch in der Freizeit damit beschäftigen“) wiederum gibt an, inwiefern die Auseinandersetzung mit dem Interessensgegenstand „primär aus ‚sachimmanenten‘ Gründen erfolgt“ (Schiefele et al. 1993, S. 337). Krapp und Prenzel (2011) folgend, wird auch die kognitiv-epistemische Komponente einbezogen, davon ausgehend, dass sich Interesse auch in einer Art Wissbegierde äußert („ich möchte mehr darüber lernen“).

#### Interesse, Kompetenz und Kontextorientierung

Das Interesse im naturwissenschaftlichen Unterricht folgt nicht nur dem Selbstzweck. Es stellt sich auch die Frage, wie Interesse mit dem Lernen (im Prozess und im Produkt) zusammenhängt. Es ist davon auszugehen, dass sich diesbezüglich positive Zusammenhänge zeigen (Krapp und Prenzel 2011), weil Interesse die Auseinandersetzung mit dem Lerninhalt fördert (Vogt 2007).

Zahlreiche Studien liefern Hinweise darauf, dass Interesse in großem Zusammenhang mit Leistungen von Lernenden, deren Motivation sowie beruflicher Orientierung in Naturwissenschaften und Technik stehen (Habig et al. 2025; Köller et al. 2006; Potvin und Hasni 2014; Schiefele et al. 1993). Das Lernen in sogenannten Kontexten (Kontextorientierung) soll dabei u. a. einen Beitrag dazu leisten, dem Interessensabfall bei Schüler:innen in den Naturwissenschaften entgegenzuwirken (Schmid 2023; Christidou 2011; Krapp und Prenzel 2011; Löffler et al. 2018; Mokhonko 2016; Potvin und Hasni 2014). Der Begriff Kontext bezeichnet eine meist außerfachliche Situation, die als Ausgangspunkt für die Erarbeitung des fachlichen Inhalts genutzt wird (Gilbert 2006; Viljaranta et al. 2014). Metastudien zu kontextbasiertem Lernen in den Naturwissenschaften von Bennett et al. (2007) und Ültay und Çalık (2012) konnten in fast allen Studien positive Effekte zu den affektiven Merkmalen – wie Einstellungen, Interesse und Enthusiasmus – und heterogene Resultate bei kognitiven Faktoren – u. a. Lernleistungen – identifizieren. In Bezug auf gestufte Hilfen fanden Kleinert et al. (2023) beispielsweise, dass der Einsatz gestufter Hilfen beim biologischen Experimentieren in positivem Zusammenhang mit der wertbezogenen Komponente des situativen Interesses steht, und zur Steigerung der wahrgenommenen Kompetenz beim biologischen Experimentieren führt.

#### Interesse, Zielorientierung und Fähigkeitsselbstkonzept

Eine Theorie, die sich mit dem individuellen Verhalten von Lernenden in Lern- und Leistungssituationen befasst, ist die der Zielorientierung (ZO; Köller und Schiefele 2010). Dabei geht man davon aus, dass die Motivation und die Herangehensweie von Lernenden, sich mit einer akademischen Aufgabe auseinander zu setzen, von ihrer innernen Zielorientierung abhängt: einer Lernzielorientierung und einer Leistungszielorientierung. Also entweder dem Ziel, sich selbst zu verbessern, sich Wissen anzueignen oder es zu erweitern (Lernen), oder dem Ziel, seine Fähigkeiten zu demonstrieren, die Aufgabe möglichst gut zu lösen (Leisten; Ames 1992; Elliot und Church 1997). Aus verschiedenen möglichen Ausprägungen dieser Zielorientierungen, hat sich ein trichotomes Modell bewährt (Elliot und Church 1997; Spinath und Schöne 2003), das sich aus der Lernzielorientierung (LZO), der Annäherungs-Leistungszielorientierung und der Vermeidungs-Leistungszielorientierung zusammensetzt. Letzteres beschreibt S:S, deren Ziel es ist, nicht durch schlechte Leistung oder Versagen aufzufallen. Die Zielorientierung ist mit diversen anderen individuellen Faktoren assoziiert, wie beispielsweise der intrinsischen Motivation oder dem Fähigkeitsselbstkonzept. Für das selbstregulierte Lernen und die Hilfen-Nutzung ist die Zielorientierung deshalb wichtig, weil sich eine LZO positiv auf das Hilfe-Suchen auswirkt (Aleven et al. 2003; Karabenick 2004; Spinath und Schöne 2003; Vaessen et al. 2014), wohingegen Vermeidungs-Leistungszielorientierte Hilfe meiden, da sie befürchten, dadurch als inkompetent zu gelten (Du et al. 2016). Für sie ist die Nutzung von Hilfe mehr als für andere mit Ängsten, Scham und Bedrohung für den Selbstwert verbunden (Tobias 2006). Weiterhin zeigen Studien, dass Lernzielorientierte stärker instrumentelle Hilfe in Anspruch nehmen, während Leistungszielorientierte eher nach exekutiven Hilfen, wie z. B. Lösungen, suchen (Arbreton 1998). Ziel sollte es sein, entsprechend die LZO zu fördern. Es stellt sich daher die Frage, wie das Interesse mit der Zielorientierung zusammenhängt. Interesse und LZO sollten schon aus definitorischen Gründen stark miteinander korrelieren, da eine Komponente des Interesses ist, mehr über den jeweiligen Gegenstand zu erfahren bzw. zu lernen (Krapp und Prenzel 2011). Wenn der Kompetenzerwerb das primäre Ziel ist (LZO) und nicht nur Mittel zum Zweck, liegt die Nähe zur intrinsischen Motivation bzw. dem Interesse sehr nahe (Köller und Schiefele 2010; Krapp 1999; Rheinberg 2010). Van Yperen (2003) konnte einen moderierenden Effekt von Zielorientierungen auf die Beziehung zwischen Interesse und Performanz feststellen, wohingegen Horvath et al. (2006) ihre entsprechende Hypothese nicht belegen konnten. Für Hausaufgaben in Mathematik konnte bspw. der Zusammenhang von Interesse mit der LZO und damit mit der Leistung gezeigt werden (Xu 2021).

Auch das Fähigkeitsselbstkonzept (FSK) spielt beim Lernen eine wichtige Rolle. Es ist die subjektive Einschätzung der eigenen Fähigkeit (Stiensmeier-Pelster und Schöne 2008). Es liegt anderen motivationalen Faktoren (z. B. Zielorientierungen und Kausalattributionen; Köller und Schiefele 2010) zugrunde. Entsprechend kann das FSK das Hilfen-Nutzungs-Verhalten im selbstregulierten Lernen beeinflussen (Stiensmeier-Pelster und Schwinger 2007). Interesse und FSK sind eng miteinander verbunden (Eccles und Wigfield 2002; Köller et al. 2006). Damerau (2012) hat sich konkret mit dem FSK beim Experimentieren beschäftigt und stellt in Anlehnung an Krapp und Ryan (2002) fest, dass es zwar eine notwendige, aber keine hinreichende Bedingung der Interessenentwicklung sei, und beschreibt durchgehend positive Zusammenhänge. Für Mathematik konnte gezeigt werden, dass frühere Leistungen das FSK beeinflussen, und dies den Zusammenhang zwischen Leistung und Interesse vermittelt. Weiterhin ergab eine Studie an Statistikstudierenden, dass sowohl Interesse als auch FSK signifikante Prädiktoren für den Lernerfolg sind und dass das FSK die Beziehung zwischen Interesse und Leistung teilweise vermittelt (Hazaimehl et al. 2023). Es lässt sich entsprechend ein reziproker Zusammenhang vermuten, wie auch die Studie von Marsh et al. (2005) aufzeigt. Jansen et al. (2014) fanden zudem Unterschiede zwischen den drei naturwissenschaftlichen Fächern, die zeigen, dass Schülerinnen in Chemie und Physik ein geringeres Selbstkonzept haben als Schüler. Diese geschlechtsbezogenen Disparitäten können auf geschlechtsstereotype Prägungen zurückgeführt werden (u. a. Kessels und Heyder 2018).

#### Interesse und Geschlecht

Geschlechtsstereotypen sind Teil einer kollektiv sozial geteilten Annahme, welche Einstellungen zu weiblichen und männlichen Rollen sowie weiblichen und männlichen Berufen und der Selbstwahrnehmung umfasst (Makarova et al. 2019). Solche Überzeugungen können motivationale Tendenzen wie Interesse, Zielorientierung und das Fähigkeitsselbstkonzept beeinflussen. Je stärker ein Fach bzw. eine Tätigkeit mit dem anderen Geschlecht assoziiert wird, desto niedriger werden das eigene Fähigkeitsselbstkonzept und die eigenen Leistungen eingeschätzt und desto kleiner wird das Interesse, sich mit diesem Fach bzw. dieser Tätigkeit auseinanderzusetzen (Kessels und Heyder 2018), was sich auf das Lernverhalten, und die Qualität der Lernergebnisse auswirkt (Kessels und Heyder 2018; Lohaus et al. 2010; Makarova et al. 2019; Schiefele und Schreyer 1994). Geschlechtsbezogene Disparitäten in Bezug auf das Interesse an naturwissenschaftlichen Fächern sowie Themengebieten sind gut belegt (bspw. OECD 2016). Je nach Fach oder Thema sind diese unterschiedlich groß und in unterschiedliche Richtung ausgeprägt. Jungen interessieren sich insbesondere für Physik und wertschätzen dieses Fach mehr, während sie im Vergleich zu den Mädchen der Biologie ein geringeres Interesse sowie Wertschätzung entgegenbringen (Elster 2010; Habig et al. 2025; Kessels und Heyder 2018; Koul et al. 2012). Auch in Chemie wiesen die Jungen in der ROSE-Studie ein signifikant höheres Interesse auf als die Mädchen (Elster 2010). Für das Experimentieren fasst Damerau (2012) zusammen, dass Mädchen ein größeres Interesse daran zeigen, sich theoretisch mit biologischen Themen zu beschäftigen, beispielsweise durch das Lesen im Biologiebuch, während Jungen mehr Interesse am praktischen Experimentieren haben (Aufdermauer und Hesse 2006). Das Interesse an der Tätigkeit Experimentieren hat somit das Potenzial, Geschlechterstereotypen entgegenzuwirken (Engeln 2006). Zudem weisen die Metastudien von Bennett et al. (2007) und Ültay und Çalık (2012) darauf hin, dass durch das kontextualisierte Lernen die geschlechtsspezifischen Unterschiede in affektiven Merkmalen, wie z. B. bei den Einstellungen, verringert werden. Daher stellt sich auch für das Planen von Experimenten die Frage, inwiefern sich Geschlechterunterschiede im Interesse finden lassen.

## Forschungsfrage und Hypothesen

Vor dem oben beschriebenen Hintergrund, der darlegt, wie die motivationalen Faktoren jeweils im Lern- und Leistungskontext wirken können, stellt sich die Frage, welche Rolle das Interesse in Zusammenhang mit den beiden weiteren motivationalen Faktoren Zielorientierung und Fähigkeitsselbstkonzept bei der Nutzung von on-demand Hilfen beim Bearbeiten einer digitalen Lernumgebung zum Planen von Experimenten spielt. Wir erwarten auf Grundlage der dargestellten Erkenntnisse Zusammenhänge zwischen Interesse, Geschlecht und Fähigkeitsselbstkonzept (H1) und vermuten, dass Interesse und Fähigkeitsselbstkonzept positiv mit der Lernzielorientierung zusammenhängen (H2). Ferner gehen wir davon aus, dass die Lernzielorientierung in positivem Zusammenhang mit der Nutzung instrumenteller Hilfen (Hinweise und Beispiele) und in negativen Zusammenhang mit der Nutzung exekutiver Hilfen (Lösungen) stehen (H3) und insbesondere instrumentelle Hilfen in positivem Zusammenhang mit dem Lernzuwachs stehen (H4). Dabei berücksichtigen wir in unseren Hypothesen sowohl direkte als auch indirekte Zusammenhänge, was nachfolgend dargelegt wird.

## Design und Methode

**Abb. 1:**
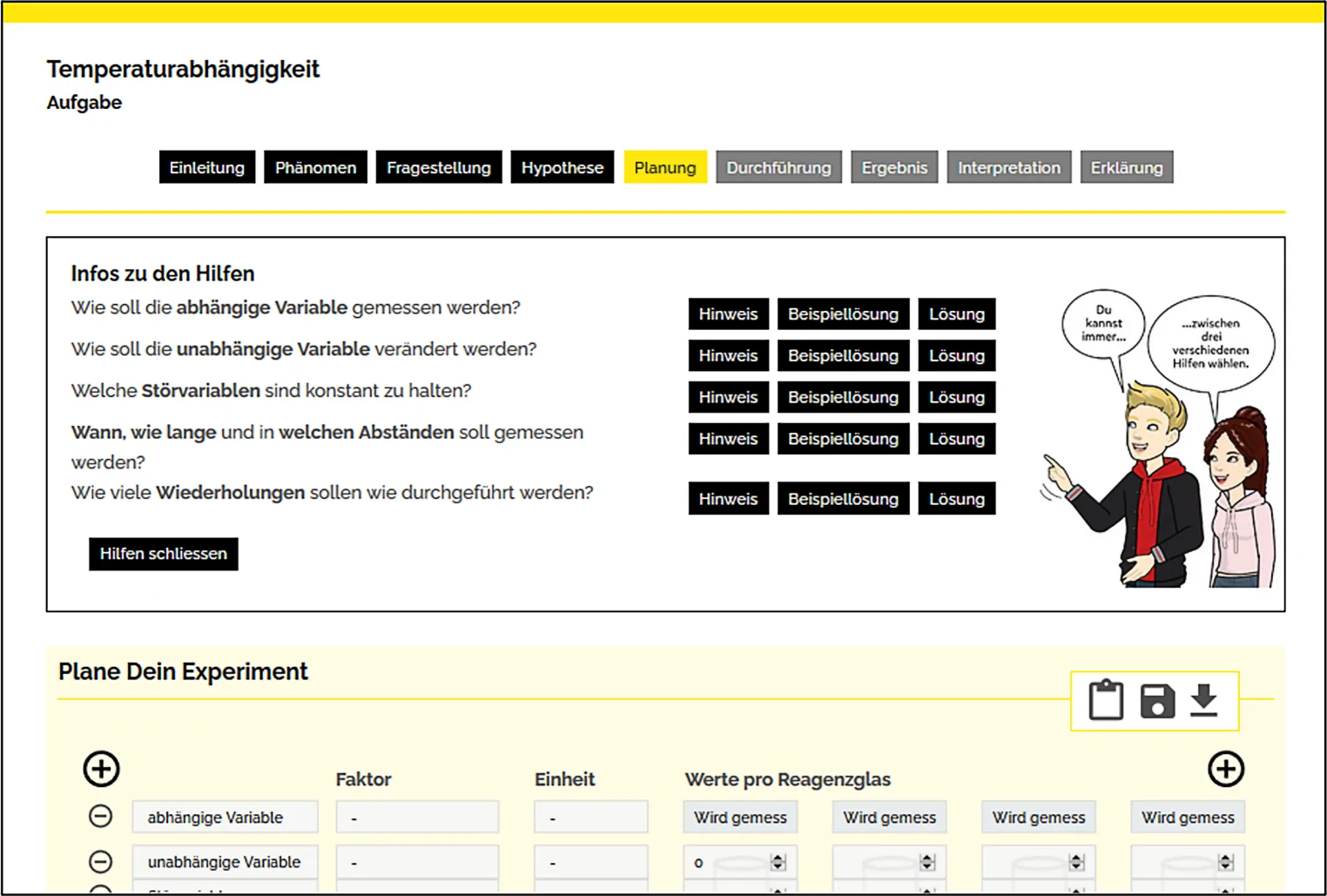
Screenshot der Aufgabe „Planung“ mit offenem Hilfefenster in der digitalen Lernumgebung „Gestufte Lernhilfen“

Um die Hypothesen zu prüfen, wurde eine Interventionsstudie im Pre-Post-Design durchgeführt. Die Lernenden bearbeiteten individuell vier digitale Lernaufgaben zum Themenbereich Enzymatik (biochemisches Thema) mit dem Ziel der Förderung der Planungskompetenz beim Experimentieren. Sie hatten die Aufgabe, im Rahmen der Lernaufgaben jeweils ein Experiment zur Prüfung einer vorgegebenen Hypothese (Temperaturabhängigkeit, pH-Wert-Abhängigkeit, Hemmung und Aktivierungsenergie) zu planen. Die Lernenden haben „Reagenzgläser“ virtuell gefüllt und Messzeiten sowie Wiederholungen angegeben. Die Lernaufgaben wurden im Sinne der kompetenzorientierten Gestaltung in einen alltagsweltlichen Kontext eingebettet (Ranzige Butter, Zellgift, Urin und Diabetes). Dazu standen ihnen gestufte Lernhilfen zur Verfügung, die die fünf relevanten Kompetenzaspekte (Arnold et al. 2018) abdeckten. Dabei konnten die Lernenden aus drei verschiedenen Formaten wählen: Hinweise, Beispiele und Lösungen. Somit standen den Lernenden pro Aufgabe 15 verschiedene Hilfen zur Verfügung, aus denen sie nach Bedarf wählen konnten (Abb. 1).

### Stichprobe

An der Studie nahmen insgesamt *N* = 678 (*n*_*female*_ = 389, *n*_*male*_ = 276, *n*_*diverse*_ = 13) Schüler:innen im Alter von 13 bis 19 Jahren teil. Das Durchschnittsalter betrug 16,0 Jahre (*SD* = 1,1 Jahre). Die Studie wurde in 31 Schulklassen (9.–12. Klasse) aus 13 Schulen in der Schweiz durchgeführt. Aufgrund von technischen Problemen und krankheitsbedingten Abwesenheiten (die Studie fand während der COVID-19-Pandemie statt) wurden von der Gesamtstichprobe nur die Daten ausgewertet, bei denen alle vier Lernaufgaben bearbeitet und eine Zeit investiert wurde, die eine sinnvolle Bearbeitung ermöglichte (> 16 min). Die vorliegende Studie basiert daher auf einer Stichprobe von *N* = 480 (*n*_*weiblich*_ = 284, *n*_*männlich*_ = 191, *n*_*divers*_ = 5). Aufgrund der geringen Fallzahl in der Kategorie divers (*n* = 5) konnte diese Kategorie nicht als eigene Ausprägung in den inferenzstatistischen Analysen modelliert werden. Für die Modellanalysen wurde daher eine binär kodierte Geschlechtsvariable verwendet (0 = weiblich, 1 = männlich).

Die Stichprobe wurde durch Anschreiben an Schulleitungen und Kontaktaufnahme mit Lehrpersonen rekrutiert. Bei Minderjährigen gaben die S:S und ihre Erziehungsberechtigten ihre schriftliche Zustimmung zur Teilnahme an der Studie. Das Forschungsprojekt wurde von der Ethikkommission der Pädagogischen Hochschule FHNW genehmigt. Nähere Informationen zur Lernumgebung sowie Screenshots finden sich auf folgender Homepage: https://www.fhnw.ch/de/ph/forschung-entwicklung/forschung/projekte/gestufte-lernhilfen.

### Instrumente

Interesse wird häufig retrospektiv erhoben und solche Maße zeigen sich als verlässlich (Krapp und Prenzel 2011). Um die Nachteile der Retrospektive (bspw. Erinnerung und Verzerrung) zu minimieren, wurde das Interesse „embedded“, also während der Durchführung erhoben (Drechsel et al. 2011). Nach jeder bearbeiteten Lernaufgabe wurden die Lernenden zum Interesse hinsichtlich des Inhaltes und des Kontextes befragt. Die Aktivitäten (Planen von Experimenten/Experimentieren), die prinzipiell für alle vier Aufgaben passend wären, wurden aus testökonomischen Gründen nur nach der ersten und letzten Aufgabe abgefragt. Die Interessensskalen des verwendeten Instruments bestanden in Anlehnung an Schiefele et al. (1993) und Krapp und Prenzel (2011) aus jeweils vier Komponenten (wertbezogene, emotionale, kognitiv-epistemische Valenz und intrinsischer Charakter) und aus je vier Items. Die Items wurden auf einer fünfstufigen Likert-Skala beantwortet (stimmt gar nicht, stimmt eher nicht, weder/noch, stimmt eher, stimmt genau). Für die interne Konsistenz wird nachfolgend McDonald’s Omega als Koeffizient berichtet, da er verzerrungsfreier als Cronbachs Alpha misst (Dunn et al. 2013). Die interne Konsistenz liegt im sehr guten Bereich (ω_Inhalt_ = 0,93, ω_Kontext_ = 0,90, ω_Aktivität_ = 0,93) (Tab. 1).

**Tab. 1:** Übersichtsschema Items Interesse.

Interessenskomponente	Inhalt (bspw. Temperaturabhängigkeit)	Kontext (bspw. Ranzigwerden von Butter)	Aktivität (Planen von Experimenten)	Aktivität (Experimentieren)
*Emotionale Valenz*	Es hat mir Spaß gemacht, mich mit [der Temperaturabhängigkeit von Enzymen/dem Ranzigwerden von Butter] zu beschäftigen		Es macht mir Spaß, [Experimente zu planen/zu experimentieren]	
*Intrinsischer Charakter*	Ich würde mich gerne auch in meiner Freizeit mit [der Temperaturabhängigkeit von Enzymen/dem Ranzigwerden von Butter] beschäftigen		Ich würde gerne auch in meiner Freizeit [Experimente planen/experimentieren]	
*Wertbezogene Valenz*	Ich finde [die Temperaturabhängigkeit von Enzymen/das Ranzigwerden von Butter] ein wichtiges Thema		Ich finde [das Planen von Experimenten/Experimentieren] ist eine wichtige Tätigkeit	
*Kognitiv-epistemische Valenz*	Ich möchte mehr über [die Temperaturabhängigkeit von Enzymen/das Ranzigwerden von Butter] erfahren		Ich möchte mehr über [das Planen von Experimenten/Experimentieren] lernen	

Die Kompetenzen im Planen von Experimenten wurde vor und nach der Bearbeitung der Lernaufgaben im geschlossenen Antwortformat erfasst. Dabei wurde das Instrument von Arnold (2015) angepasst. Das Instrument besteht aus elf Items und prüft einzelne Kompetenzaspekte (abhängige und unabhängige Variable sowie Kontrollvariablen identifizieren, Messzeiten anwenden und Versuchsanordnungen durch Wiederholungen ergänzen). Die interne Konsistenz des Tests beträgt im Pre-Test ω = 0,57 und im Post-Test von ω = 0,72.

Die persönliche LZO wurde im Rahmen des Pre-Tests erfasst. Die Skala wurde von Spinath et al. (2012) übernommen und auf das Planen von Experimenten angepasst. Sie besteht aus acht Items (bspw. „Wenn ich Experimente plane, geht es mir darum, etwas Interessantes zu lernen.“), die auf einer fünfstufigen Likert-Skala beantwortet wurden. Die interne Konsistenz liegt bei ω = 0,84.

In der vorliegenden Studie wurde das FSK in Anlehnung an die *Skalen zur Erfassung des schulischen Selbstkonzepts* (SESSKO, Schöne et al. 2012) operationalisiert. Das Instrument trennt im Gegensatz zu anderen Instrumenten klar zwischen Selbstkonzept und Selbstwert (als affektive Bewertung Spinath und Schöne 2003). Für die hier dargestellten Ergebnisse wurde auf die kriteriale Bezugsnorm zurückgegriffen und die Items auf den Studieninhalt angepasst. Im Rahmen dieser Studie wurde das FSK explizit auf das Lösen der Experimentieraufgabe in Bezug auf das Planen von Experimenten bezogen (z. B. „Wenn ich an das Lösen der Experimentieraufgabe denke, halte ich mich für begabt“). Die fünf Items wurden auf einer fünfstufigen Likert-Skala beantwortet. Die Skala wies bei der vorliegenden Stichprobe eine interne Konsistenz von ω = 0,90 auf. Um die Passung zu den Aufgaben sicher zu stellen, wurde das FSK retrospektiv im Rahmen des Post-Tests erfasst.

Bei der Frage nach dem Geschlecht standen für die Selbstauskunft drei Optionen zur Verfügung: „weiblich“, „männlich“ und „divers“. Die Wahl der Hilfen wurde im System getrackt.

### Statistische Analysen

Die Datenaufbereitung und deskriptiven Analysen erfolgten mit der Software IBM SPSS (Version 28, 2023). Für die inferenzstatistische Analyse wurde die Software RStudio (Version 04.2.764, 2024) mit dem Paket lavaan (Version 0.6-18) genutzt. Die Analysen wurden in zwei Schritten durchgeführt: Erstens wurde die deskriptive Statistik untersucht, zweitens erfolgte eine inferenzstatistische Analyse entlang der Fragestellung und Hypothesen. Zur Überprüfung der aus bestehenden, bereits validierten Skalen eingesetzten Erhebungsinstrumente und für die Erstellung von Messmodellen wurden konfirmatorische Faktorenanalysen genutzt. Um die aus der Theorie abgeleiteten Hypothesen mit den vorliegenden Daten zu prüfen und mögliche direkte als auch indirekte Zusammenhänge zu berücksichtigen, wurden Strukturgleichungsmodelle erstellt und empirisch mit χ^2^-Teststatistik geprüft. In einem ersten Schritt wurde ein vollständiges Modell mit allen möglichen Pfaden zwischen den Konstrukten inkl. den Kovarianzen zwischen den drei Lernhilfen modelliert (Modell 1). In einem weiteren Schritt wurde das vollständige Modell mit einem Alternativmodell (Modell 2) verglichen (Weiber und Sarstedt 2021). Für das Alternativmodell wurde das Modell 1 mit empirischer Begründung modifiziert, indem Pfade nicht signifikanter Zusammenhänge zwischen den Konstrukten auf Null gesetzt wurden (Modell 2): Fähigkeitsselbstkonzept zu allen drei Lernhilfen, Interesse zu allen drei Lernhilfen, Geschlecht auf Lernhilfe_Hinweise, Geschlecht auf Lernhilfe_Beispiel, Geschlecht auf Lernzuwachs, Lernzielorientierung auf Lernzuwachs. Eine Ausnahme bildeten die Kovarianzen zwischen den drei Lernhilfen, die vollständig im Modell erhalten blieben, um die Konstruktnähe abzubilden, was theoretisch begründet ist. Dabei wurden die in den Modellen angegebenen Schätzer bei nicht multivariater Normalverteilung mit robuster Standardfehlerschätzung (MLR) verwendet. Die Modellgüte wird in Zusammenhang mit den im χ2-Anpassungstest verwendeten Parametern berichtet. Die Modell-Fit-Indizes sind in Tab. 2 aufgeführt.

**Tab. 2:** Modellfit der Strukturgleichungsmodelle mit Interesse, Fähigkeitsselbstkonzept, Lernzielorientierung, Geschlecht, Hilfenutzung und Lernzuwachs (*N* = 440)

Modell	χ^2^	*df*	*p*	CFI	TLI	RMSEA	SRMR	AIC	BIC	ΔAIC	ΔBIC
Modell 1 Vollständige Modell (mit allen Pfaden zwischen Konstrukten inkl. mit Kovarianzen Hilfestellungen)	340,58	166	< 0,001	0,95	0,94	0,05	0,05	22.285,79	22.547,35	–	–
Modell 2 Modifiziertes Modell (signifikante Zusammenhänge mit Kovarianzen Hilfestellungen)	361,73	178	< 0,001	0,95	0,94	0,05	0,05	22.284,33	22.496,85	−1,46	−50,5

Zur Berechnung der Modellfits wird eine robuste Schätzung mittels „Robust Maximum Likelihood“ (*MLR*) verwendet

## Ergebnisse

Die deskriptiven Statistiken sind in Tab. 3 im Anhang dargestellt.

**Abb. 2:**
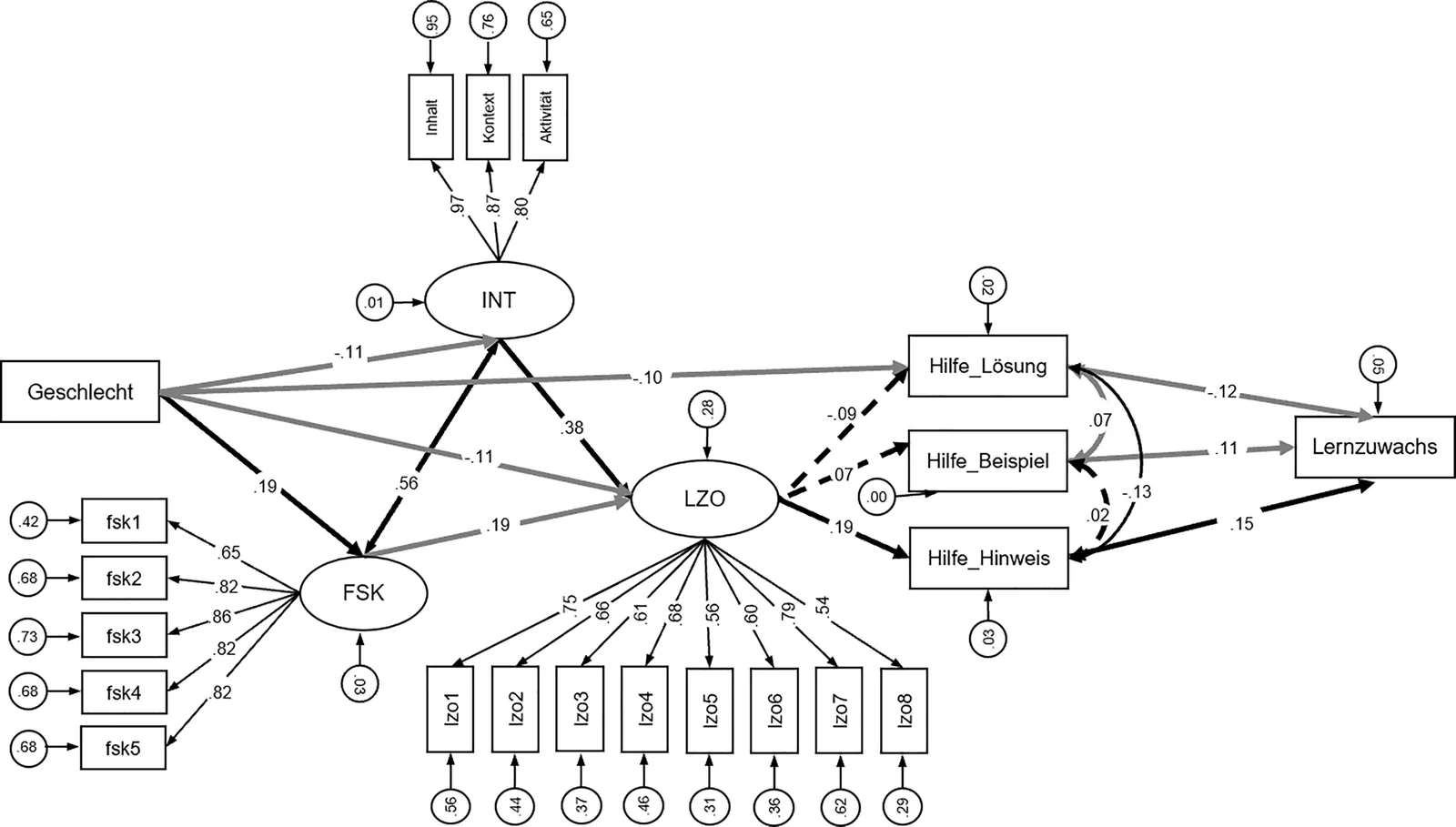
Modifiziertes Strukturgleichungsmodell 2 mit Interesse, Fähigkeitsselbstkonzept, Lernzielorientierung, Geschlecht, Hilfenutzung und Lernzuwachs (*N* = 440). *Durchgezogene Linien* zeigen hoch signifikante Beziehungen (*p* < 0,01), *graue Linien* signifikante (*p* < 0,05) und *gestrichelte Linien* nicht signifikante Beziehungen (*p* ≥ 0,05). *INT* Interesse, *FSK* Fähigkeitsselbstkonzept, *LZO* persönliche Lernzielorientierung. Geschlecht Kodierung: weiblich = 0, männlich = 1

**Abb. 3:**
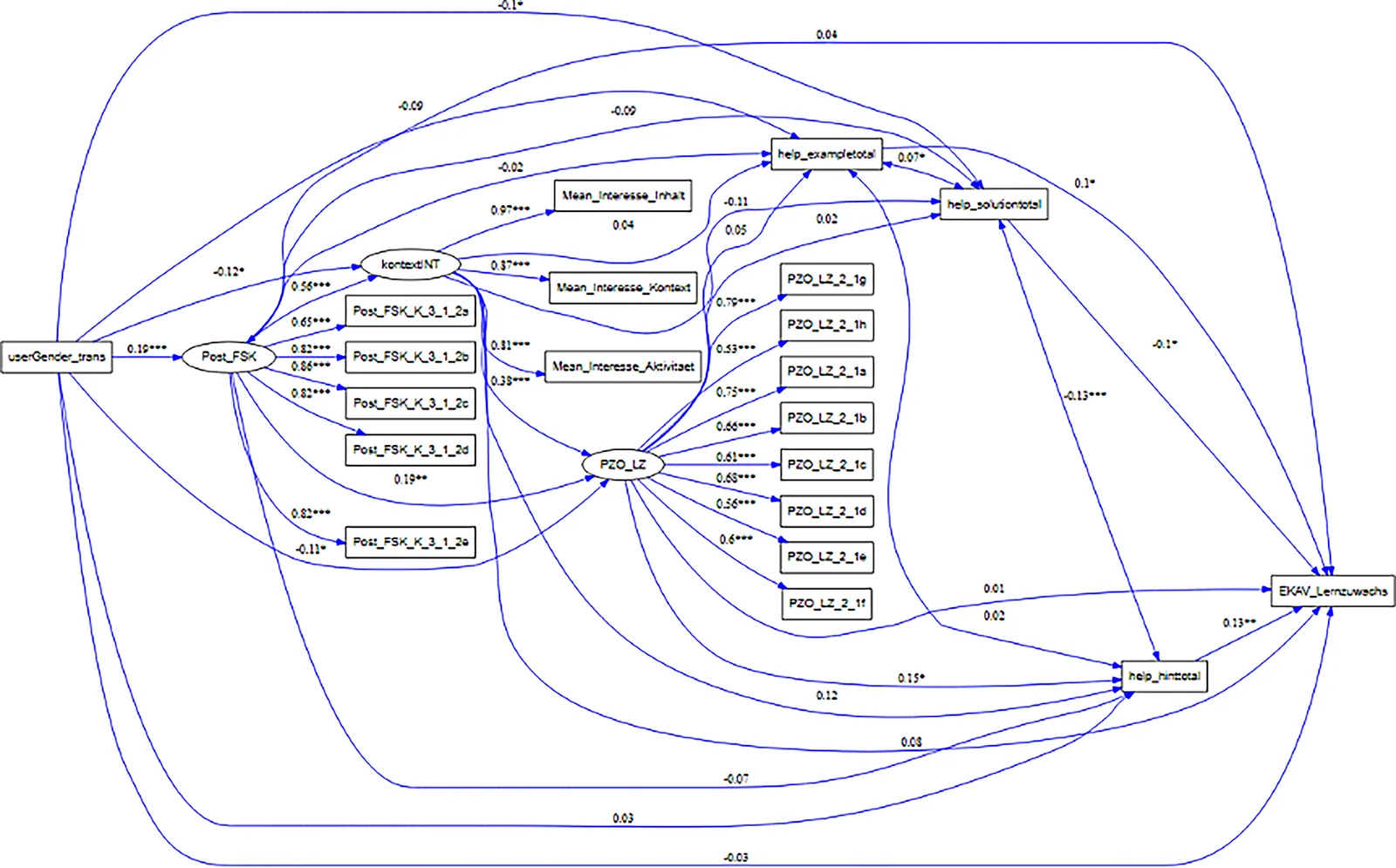
Vollständiges Strukturgleichungsmodell (Modell 1)

Nachfolgend werden in der Tab. 2 die Fit-Indizes für das vollständige Strukturgleichungsmodell (Modell 1, siehe Abb. 3 im Anhang) und das Alternativmodell (Modell 2, siehe Abb. 2) aufgeführt.

Modell 2 und Modell 1 unterscheiden sich hinsichtlich Fit-Indizes nicht signifikant, wie ein χ^2^-Differenzentest (Likelihood Ratio-Test) aufzeigt (Δχ^2^ = 21,00, Δ*df* = 12, *p* > 0,05). Die absolute Differenz des CFI beider Modelle beträgt ≤ 0,002 (Modell 1: CFI = 0,953, Modell 2: CFI = 0,951, ΔCFI^2^ = 0,002; Meade et al. 2008). Bei Vergleichen von Strukturgleichungsmodellen wurden zudem die Differenzen der AIC- und BIC-Werte pro Modell betrachtet. Tiefe Werte sprechen für eine bessere Modellpassung (Schermelleh-Engel et al. 2003). Der AIC- und BIC-Wert liegen im Modellvergleich bei Modell 2 tiefer und der *p*-Wert unterscheidet sich nicht, sodass Modell 2 als besseres Modell angenommen werden kann (Schermelleh-Engel et al. 2003).

Das Modell 2 kann gemäß den passenden Modellfit-Werten in Tab. 2 angenommen werden (Hu und Bentler 1999). Der χ^2^-Test reagiert empfindlich auf die Stichprobengröße, sodass bei großen Stichproben ein angemessenes Modell fälschlicherweise ablehnt bzw. signifikant wird (Kyriazos 2018). Entsprechend wurde auch in den Modellschätzungen ein signifikanter χ^2^-Test bei der vorliegenden Stichprobe (*N* > 400) nicht als Modellablehnung gewertet. Nachfolgend werden entlang des Modells in der Abb. 2 bedeutsame, signifikante Einflüsse erläutert. Das Geschlecht (Kodierung 0 = weiblich, 1 = männlich) weist einen bedeutsamen Zusammenhang mit FSK (β = 0,19, *p* < 0,001) sowie einen signifikanten, tendenziell negativen Zusammenhang mit Interesse (β = −0,11, *p* < 0,05) auf. Zudem zeigt sich beim Geschlecht ein signifikant negativer Zusammenhang auf die Lernzielorientierung (β = −0,11, *p* < 0,05) und die Nutzung von Lösungen als Hilfen (β = −0,10, *p* < 0,05). Die Kovarianz des Fähigkeitsselbstkonzepts und des Interesses impliziert einen hohen, positiven Zusammenhang (φ = 0,56, *p* < 0,001) (H1). Die standardisierten Pfadkoeffizienten des direkten Effekts des Interesses (β = 0,38, *p* < 0,001) und des FSK (β = 0,19, *p* < 0,05) erweisen sich als unterschiedlich bedeutsam auf die LZO: Interesse und FSK zeigen einen direkten positiven Zusammenhang mit der LZO (H2). Die LZO ihrerseits weist einen bedeutsamen Einfluss auf die Nutzung von Hinweisen beim Lernen (β = 0,19, *p* < 0,001) auf, jedoch nicht auf die Beispiele und Lösungen. Die Anzahl genutzter Hinweise zeigt einen signifikanten Zusammenhang mit dem Lernzuwachs auf (β = 0,15, *p* < 0,01) (H4). Des Weiteren zeigt sich, dass die Nutzung von Lösungen tendenziell negativ (β = −0,12, *p* < 0,05) mit dem Lernzuwachs und die Nutzung von Beispielen signifikant positiv (β = 0,11, *p* < 0,05) zusammenhängen (H4).

## Diskussion

Das Modell 2 (vgl. Abb. 2) zeigt, dass das Geschlecht sowohl mit dem FSK als auch mit dem Interesse zusammenhängt. Die in Tab. 3 im Anhang dargestellten Interkorrelationen mit der binär kodierten Geschlechtsvariable sowie das Strukturgleichungsmodell (vgl. Abb. 2) deuten darauf hin, dass männliche Teilnehmende ein leicht positiveres Fähigkeitsselbstkonzept, aber ein geringeres Interesse an den Lernaufgaben berichten als weibliche Teilnehmende. Das FSK und das Interesse stehen in starkem positivem Zusammenhang miteinander und das Interesse sowie das FSK wiederum mit der LZO, wobei der Zusammenhang zum Interesse stärker ist. Dies spricht dafür, die Hypothese 1 auf Grundlage der vorliegenden Daten anzunehmen. Im Modell wird – entlang der breit postulierten reziproken Wirkung von Interesse und Selbstkonzept (Marsh et al. 2005) – ersichtlich, dass das Interesse sowohl in indirekter Beziehung, gemittelt über das FSK, als auch in direkter Beziehung zur Zielorientierung steht. Dies wird als Beleg für H2 angesehen. Insgesamt kann anhand dieser Studie gezeigt werden, dass das Interesse in positivem Zusammenhang mit der LZO und damit auch mit der Nutzung von Lernhilfen (insbesondere der lernförderlichen Hinweise) steht. H3 konnte jedoch nur in Teilen bestätigt werden. Zwar stehen LZO und die Nutzung von Hinweisen in positivem Zusammenhang, der erwartete positive Zusammenhang zu den Beispielen und ein negativer Zusammenhang zu den Lösungen konnte in der Form empirisch nicht fundiert werden. Dies kann darauf zurückgeführt werden, dass Lösungen sowohl exekutiv als auch instrumentell und somit unterschiedlich lernförderlich genutzt werden können. So kann die Lösung ohne großen eigenen Aufwand genutzt werden, um die Aufgabe möglichst schnell zu lösen (Gaming the System; Baker et al. 2008a, b). Andererseits können die Lösungen aber auch genutzt werden, nachdem die Aufgabe möglichst selbständig gelöst wurde, um das Ergebnis zu überprüfen. Darauf weisen auch die Kovarianzen der Hilfen hin. Während Hinweise und Lösungen negativ zusammenhängen, spricht dies eher für eine exekutive Nutzung. Jedoch hängen die Beispiele mit den Lösungen leicht positiv zusammen. Hier sind nähere Analysen notwendig, um die konkrete Nutzung der Lösungen zu untersuchen. Es ist zu vermuten, dass unterschiedliche Nutzungsmuster hinter den Daten stecken, die durch stärker qualitative Daten (warum wurde welche Hilfe genutzt) aufgedeckt werden könnten. Die LZO wiederum hängt positiv mit der Nutzung von Hinweisen zusammen und Hinweisnutzung positiv mit dem Lernzuwachs. Ebenso steht die Ansicht von Beispielen in positivem Zusammenhang mit dem Lernzuwachs, wohingegen das Hinzuziehen von Lösungen im negativen Zusammenhang mit dem Lernzuwachs stehen. Auch hier zeigt sich das Muster der Lösungen als stärker exekutive Hilfe im Vergleich zu den Hinweisen und Beispielen als instrumentelle Hilfen. Somit konnten in der vorliegenden Kohorte empirisch Belege für H4 gefunden werden. Spannend ist in diesem Zusammenhang auch der direkte Zusammenhang zwischen Geschlecht und der Nutzung von Lösungen. Männliche Teilnehmende scheinen weniger Lösungen zu nutzen als weibliche. Auch hier sind stärker qualitative Untersuchungen notwendig, um die Nutzungsmotive genauer zu analysieren.

Die Studie konnte den Stand der Forschung insofern bestätigen, dass instrumentelle Hilfen (Hinweis und Beispiele) sich als lernförderlich erwiesen (Kleinert et al. 2023) und exekutive Hilfen (Lösungen) eher als lernhinderlich (Nelson-Le Gall 1985; Schmidt-Borcherding et al. 2013; Schmidt-Weigand et al. 2008). Ebenso erwartungskonform hat sich gezeigt, dass die LZO lernförderlich ist, und positiv mit dem FSK zusammenhängt (Köller und Schiefele 2010).

Im Zentrum dieses Beitrags stand die Rolle des Interesses bei der Nutzung von Lernhilfen zur Förderung von Experimentierkompetenzen in einer digitalen Lernumgebung. Diesbezüglich zeigt sich, dass das Interesse stark mit dem FSK zusammenhängt und sowohl direkt als auch indirekt über das FSK auf die LZO wirkt. Dies wiederum hat einen Zusammenhang mit der Hilfennutzung und damit zum Lernzuwachs, was zu den theoretischen Annahmen passt, dass Interesse und LZO stark zusammenhängen (Krapp 1999; Rheinberg 2010; Köller und Schiefele 2010). Die vorliegende Studie zeigt anhand der theoretischen und empirischen Prüfung die Bedeutsamkeit des Interessenskonstrukts beim Planen von Experimenten im naturwissenschaftlichen Unterricht anhand digitaler Lernumgebung auf. Dabei wird Interesse anhand der unterschiedlichen Kontexte situational hervorgerufen (Schmid 2023; Zügge 2022) und ermöglicht einen positiven Einfluss auf die persönliche Lernzielorientierung und den Lernzuwachs (Xu 2021). Dies stützt den in Meta‑/Reviewstudien aufgezeigten positiven Effekt von Kontexten, da diese prinzipiell über das Interesse lernwirksam werden (Habig et al. 2025; Ültay und Çalık 2012).

Spannend ist dabei auch, dass Schüler zwar ein höheres FSK aufweisen, jedoch Schülerinnen höheres Interesse. Dies entspricht dem Stand der Forschung, wenn man berücksichtigt, dass Enzymatik ein biochemisches Thema ist (Damerau 2012; Jansen et al. 2014; siehe Abschnitt „Interesse und Geschlecht“). So kann ggf. das mangelnde FSK durch eine stärkere LZO ausgeglichen und entsprechend lernförderliche Hilfennutzungsmuster getriggert werden. Dadurch kommt es dann vermutlich eher zu einem Kompetenzerleben, was sich wiederum positiv auf das FSK auswirken könnte. Entsprechend konnte in dieser Arbeit ein motivationaler Pfad beschrieben werden, der erklärt, wie insbesondere weibliche Teilnehmende in naturwissenschaftlichen Aufgaben gefördert werden können.

## Limitationen und Ausblick

Die vorliegende Studie gibt erstmals Einblick in die Rolle des Interesses in der Auseinandersetzung mit einer (digitalen) Lernumgebung zum Experimentieren mit gestuften Lernhilfen. Diesbezüglich ist die Studie eher explorativ und liefert Hinweise für weitere experimentelle Untersuchungen. Entsprechend können durch diese Studie keine Kausalzusammenhänge untersucht werden. Weiterhin erlaubt die Studie nur Aussagen über einen sehr engen Themenbereich der Enzymatik und müsste sicherlich auch in anderen Themen getestet werden. Dennoch gibt die Studie wertvolle Informationen über das Lernen in der Lernumgebung und der Nutzung gestufter Lernhilfen und liefert damit einen Beitrag zur Schließung der Forschungslücke.

Wir konnten zeigen, dass Interesse eine große Bedeutung für die LZO und damit auch für die lernförderliche Hilfenwahl hat und konnten das Interesse in strukturellen Zusammenhang mit weiteren motivationalen Konstrukten zur Hilfennutzung und entsprechend dem Lernzuwachs bringen. Somit liegen Hinweise vor, die nahelegen, dass eine LZO nicht nur über motivationale Faktoren, wie bspw. individuelle Bezugsnormorientierung (Schöne et al. 2004) und Sense of Achievement für ein adäquates FSK (Schütte et al. 2017) gefördert werden kann. Vielmehr haben wir auch einen fachlichen Hebel bestätigt, der an beiden Punkten ansetzt: das Interesse. So könnte bspw. bei höherem Interesse auch eine lernförderliche Hilfenwahl und somit größerer Lernzuwachs getriggert werden. Nähere Analysen darüber, welche Themen und Kontexte jeweils geeignet sind, müssten in der Breite über verschiedene Themen durchgeführt werden. Insgesamt scheint hier eine Möglichkeit zu liegen, insbesondere die Mädchen zu fördern und damit ihr tendenziell geringeres FSK durch gesteigerte LZO zu kompensieren.

## Data Availability

Die dieser Arbeit zugrunde liegenden anonymisierten Daten sind auf begründete Anfrage bei der Korrespondenzautorin erhältlich, soweit dies datenschutzrechtlich und gemäss den Einwilligungen der Teilnehmenden zulässig ist.

## Anhang

​

**Tab. 3:** Deskriptive Statistik und Interkorrelationen.

		Geschlecht	Fähigkeitsselbstkonzept	Interesse				Lernzielorientierung	Anzahl genutzte Hilfen			Lernzuwachs Kompetenz
				Inhalt	Kontext	Aktivität	Gesamt		Hinweise	Lösungen	Beispiellösungen	
Geschlecht (m = 1; f = 0)		0,40 (0,49)	–	–	–	–	–	–	–	–	–	–
Fähigkeitsselbstkonzept		0,132^**^	1,86 (0,81)	–	–	–	–	–	–	–	–	–
Interesse	Inhalt	0,079	0,473^***^	1,65 (0,77)	–	–	–	–	–	–	–	–
	Kontext	0,030	0,401^***^	0,891^***^	1,62 (0,72)	–	–	–	–	–	–	–
	Aktivität	0,106^*^	0,498^***^	0,785^***^	0,713^***^	2,02 (0,79)	–	–	–	–	–	–
	Gesamt	0,078	0,494^***^	0,959^***^	0,930^***^	0,900^***^	1,76 (0,71)	–	–	–	–	–
Lernzielorientierung		0,031	0,319^***^	0,448^***^	0,410^***^	0,520^***^	0,496^***^	3,04 (0,61)	–	–	–	–
Anzahl Hilfen	Hinweise	−0,078	0,057	0,126^**^	0,135^**^	0,143^**^	0,145^**^	0,158^**^	1,99 (2,19)	–	–	–
	Lösungen	−0,049	−0,144^**^	−0,133^**^	−0,102^*^	−0,135^**^	−0,133^**^	−0,071	−0,144^**^	2,59 (3,81)	–	–
	Beispiellösungen	−0,057	−0,004	0,054	0,076	0,052	0,065	0,054	0,032	0,075	0,68 (1,26)	–
Lernzuwachs Kompetenz		−0,049	0,095^*^	0,129^**^	0,142^**^	0,142^**^	0,148^**^	0,090	0,183^***^	−0,133^**^	0,106^*^	1,03 (2,42)

Mittelwerte und Standardabweichungen (in der Diagonale)

^***^ Die Korrelation (Pearson) ist signifikant auf dem Niveau von 0,001 (2-tailed)

^**^ Die Korrelation (Pearson) ist auf dem 0,01-Niveau signifikant (2-tailed)

^*^ Die Korrelation ist auf dem 0,05-Niveau signifikant (2-tailed)
